# rTMS efficacy in major depression disorder: comparing two reviews

**DOI:** 10.1192/j.eurpsy.2023.1790

**Published:** 2023-07-19

**Authors:** S. Yasmine, B. A. Hanen, B. O. Fatma, A. Amal, K. Emira, M. Leila

**Affiliations:** RAZI hospital, TUNIS, Tunisia

## Abstract

**Introduction:**

Transcranial magnetic stimulation (TMS)is perhaps the most popular of the newbrain stimulation techniques because its clinical effects are produced without the need for a craniotomy (as with deep brainstimulation (DBS)) or seizure induction(as with electroconvulsive therapy (ECT)).Recently ,TMS is typically used to improve depression symptoms when other depression treatments haven’t been effective.

**Objectives:**

This is a review that discuss the efficacy of the antidepressant effect of TMS.

**Methods:**

A narrative review was conducted basedon a search in pubmed using thekeywords: “rTMS” and “depression”.

**Results:**

One of the reviews studied proved the efficacy of rtms in the treatment of depressionbut there is still a clinical need for the complementary use of antidepressants.Although rTMS is more expensive than conventional antidepressants, it remains more interesting for patients who have not found benefits with pharmacological treatments.The other review also demonstrated the antidepressant effect of rTMS and that this effect, once completed, appears to be as long-lasting as that of antidepressants. TMS is also a promising new therapy and a powerful research tool. The body of TMS literature suggests that daily, left prefrontal TMS for 3–6 weeks has antidepressanteffects that are clinically meaningful (30% remission), with low side effects and nodrug-drug interactions. Furthermore, TMS shows promise in several other psychiatricdisorders, particularly treating acute and chronic pain.

**Conclusions:**

Even though The Food and drug administration (FDA) has accorded RTMS’ initial clearance of the first device in 2008 ,additional researches are still needed.The TMS coil location, stimulation intensity and frequency, and dosing strategy have to be more precise for better results.

**Disclosure of Interest:**

None Declared

